# Supplementing *N*-carbamoylglutamate in late gestation increases newborn calf weight by enhanced placental expression of mTOR and angiogenesis factor genes in dairy cows

**DOI:** 10.1016/j.aninu.2021.05.007

**Published:** 2021-09-28

**Authors:** Fengfei Gu, Luyi Jiang, Linyu Xie, Diming Wang, Fengqi Zhao, Jianxin Liu

**Affiliations:** a Institute of Dairy Science, College of Animal Sciences, Zhejiang University, Hangzhou, 310058, China; b Department of Animal and Veterinary Sciences, University of Vermont, Burlington, VT 05405, USA

**Keywords:** *N*-Carbamoylglutamate, Metabolomics, Newborn calf weight, Nutrient transporter, Placentome, Transition dairy cows

## Abstract

The objective of this study was to investigate whether supplementation with *N*-carbamoylglutamate (NCG) to cows during late gestation alters uteroplacental tissue nutrient transporters, calf metabolism and newborn weight. Thirty multiparous Chinese Holstein cows were used in a randomized complete block design experiment. During the last 28 d of pregnancy, cows were fed a diet without (CON) or with NCG (20 g/d per cow). The body weight of calves was weighed immediately after birth. Placentome samples were collected at parturition and used to assess mRNA expression of genes involved in transport of arginine, glucose, fatty acid and angiogenesis factors, as well as the mammalian target of rapamycin (mTOR) pathway. Blood samples of calves before colostrum consumption were also collected for the detection of plasma parameters, amino acids (AA) and metabolomics analysis. The newborn weight (*P* = 0.02) and plasma Arg concentration of NCG-calves was significantly higher (*P* = 0.05) than that of CON-calves, and the plasma concentrations of urea nitrogen tended to be lower (*P* = 0.10) in the NCG group. The mRNA abundance of genes involved in glucose transport (solute carrier family 2 member 3 [*SLC2A3*], *P* < 0.01), angiogenesis (nitric oxide synthase 3 [*NOS3*], *P* = 0.02), and mTOR pathway (serine/threonine-protein kinase 1 [*AKT1*], *P* = 0.10; eukaryotic translation initiation factor 4B pseudogene 1 [*EIF4BP1*], *P* = 0.08; *EIF4EBP2*, *P* = 0.04; and E74-like factor 2 [*ELF2*], *P* = 0.03) was upregulated in the placentome of NCG-supplemented cows. In addition, 17 metabolites were significantly different in the placentome of NCG-supplemented cows compared to non-supplemented cows, and these metabolites are mainly involved in arginine and proline metabolism, alanine, aspartate and glutamate metabolism, and citrate cycle. In summary, the increased body weight of newborn calves from the NCG supplemented dairy cows may be attributed to the increased angiogenesis and uteroplacental nutrient transport and to the activated mTOR signal pathway, which may result in the increased nutrient supply to the fetus, and improved AA metabolism and urea cycle of the fetus.

## 1 Introduction

Calf birth weight is strongly associated with calf health, growth, and future productivity (Johanson and Berger, 2003). In dairy cows, the final trimester of gestation is characterized by marked fetal growth, and abundant nutrients are required to ensure adequate fetal growth during this period (NRC, 2001). Placental development and functions play important roles in the growth and development of the fetus. Placental angiogenesis supports the required blood flow and nutrient transport to the fetal side; thus, increased angiogenesis could improve fetal growth (Liu et al., 2012). In addition, Batistel et al. (2017) reported that the increased newborn birth weight was strongly associated with the increased mRNA abundance of genes encoding amino acid (AA), glucose, and fatty acid transporters in the placenta. In addition to the indispensable function of delivering nutrients essential for fetal growth, placental tissue (as do other mammalian tissues) has an array of nutrient-sensing signaling pathways, such as the mammalian target of rapamycin (mTOR) complex (Jansson et al., 2013). The mTOR pathway can alter the activity of placental nutrient transporters, such as AA transporters (Roos et al., 2009).

Arg is a semi-essential AA with various physiological functions (Wu et al., 2009). Many studies have shown that Arg regulates placental function and has great impacts on fetal survival, growth and development (Santana et al., 2014; Wu et al., 2013). Dietary supplementation of Arg products can prevent fetal growth restriction in humans (Xiao and Li, 2005) and rats (Vosatka et al., 2005). Supplementation of rumen-protected Arg has been shown to improve placental development in ewes (Zhang et al., 2016) and promote intestinal absorption of AA by regulating the mTOR signaling pathway (Zhang et al., 2019). However, the rapid rumen degradation and high price of Arg limit its utilization in dairy cows. Alternatively, *N*-carbamoylglutamate (NCG), a structural analog of *N*-acetylglutamate that is essential for endogenous Arg synthesis (Gessler et al., 2010), has unique advantages, including low rumen degradability, low cost, and a lack of negative effects on intestinal absorption of dietary His and Lys (Chacher et al., 2012). Cai et al. (2018) reported that NCG has great potential to improve pregnancy outcomes in sows. In gilts, maternal NCG supply during early pregnancy improves embryonic survival and development through modulation of endometrial proteomes (Zhang et al., 2014; Zhu et al., 2015).

Recently, we reported that supplemental NCG in transitional dairy cows could increase the supply of Arg and improve the health and production performance of dairy cows (Gu et al., 2021). In this companion study, we investigated the effects of supplementing NCG to dairy cows during late pregnancy on fetal growth, newborn calf weight, expression of nutrient transporters in uteroplacental tissue, and calf blood metabolites.

## 2 Materials and methods

### 2.1 Animals and experimental design

The experimental procedures used in this study were approved by the Animal Care Committee of Zhejiang University (Hangzhou, China) and were conducted in accordance with the university's guidelines for animal research. The details for the experimental design were described previously (Gu et al., 2021). In brief, 30 multiparous Chinese Holstein dairy cows with similar body weight (BW; 657 kg, SD = 58) at wk 4 before parturition were assigned to 15 blocks according to parity, 305-d milk yield (8,692 kg, SD = 607) of the previous lactation, BW, and body scores. The first week (wk 4 before parturition) was used as the adaptive period. The dairy cows were then randomly allocated into 2 treatments: basal diet without (CON) or supplemented with 20 g/d NCG (NCG) (Purity >97%; Beijing Animore Sci. & Tech. Co., Ltd., Beijing, China). The parities were 2.80 (SD = 0.86) and 2.67 (SD = 0.81) in the CON and NCG groups, respectively. The chemical compositions of the diets are as described previously (Gu et al., 2021). Throughout the trial period, cows were housed in a barn with individual tie stalls and had free access to water. The NCG was supplemented once per d at 14:00 by top-dressing it on the total mixed ration for individual cows. Newborn calves were weighed immediately after parturition and before colostrum consumption.

### 2.2 Blood sample collection and analyses

Blood samples from each newborn calf were collected from the jugular vein, deposited into 10 mL tubes containing an anticoagulant (heparin lithium) before colostrum consumption, and centrifuged at 3,000 × *g* for 15 min at 4 °C for collection of plasma. The plasma was then frozen at −80 °C until subsequent analysis. A subsample of each plasma sample was used to analyze biochemical variables, including glucose (ZH2079T), total protein (ZH2012G), blood urea nitrogen (ZH2017S), non-esterified fatty acids (ZH2045Z), β-hydroxybutyrate (ZH2029T), cholesterol (ZH2040Z), triglyceride (ZH2039Z), total bilirubin (ZH2007G), albumin (ZH2013G), and globulin using an AutoAnalyzer 7,020 instrument (Hitachi High-technologies Corporation, Tokyo, Japan) with colorimetric commercial kits (Ningbo Medical System Biotechnology Co., Ltd., Ningbo, China). The globulin was obtained by subtracting albumin from total protein; thus all globulins were included in the measurement. The plasma AA compositions were determined as described previously (Gu et al., 2021). Briefly, ice-cold sulfosalicylic acid (50 g/L) was added to the plasma (1:4, vol/vol) to precipitate the protein. The sample was then centrifuged at 8,320 × *g* for 30 min at 4 °C. The supernatant was collected, filtered through 0.45 μm and then 0.22 μm nylon syringe filters (Fisher Scientific, Pittsburgh, PA), and then analyzed with Automatic AA Analyzer (Hitachi High-technologies Corporation, Tokyo, Japan). Six plasma subsamples in each group were collected based on the following criteria: same parities of the dairy cows (parity = 2), similar number of days from the date when the cows began receiving NCG to the calving day (20.2 d, SD = 0.75), and calf sex (female) and used to analyze the metabolome profiles with GC–MS (Wu et al., 2018).

### 2.3 Placentome collection and analyses

The placentome samples were collected following the methods reported by Batistel et al. (2017). Briefly, after natural delivery of the calf (within 2 h), the placenta was rinsed with physiologic saline, and 4 to 6 placentomes from the central area of the placenta were dissected, pooled after cleaning with physiologic saline, and stored at −80 °C for later analysis. Six placentas were selected from each group for gene expression analysis. The criteria of animal selection were as follows: same parity, body condition, and block of the cows, similar numbers of days from the date when the cows began receiving NCG to the calving day (20.2 d, SD = 0.75), similar times when the placenta was released after parturition (within 2 h), and same calf sex (female).

Total RNA from the placentome was extracted with TRIzol reagent according to the manufacturer's instructions (Aidlab Inc.; Code: RN03). The concentration and purity of the total RNA were measured using a NanoDrop ND-1000 Spectrophotometer (NanoDrop Technologies Inc., Wilmington, DE, USA). The total RNA of the placentome was reverse transcribed to cDNA using the PrimeScript 1st Strand cDNA Synthesis Kit (TOYOBO, Osaka, Japan; Code: FSQ-101). Quantitative real-time PCR (qRT-PCR) was performed with the 2 × SYBR Premix Ex Taq kit (Aidlab Inc., Beijing, China; Code No. PC5902) and the Applied Biosystems 7,500 system (Foster City, CA). The PCR conditions were set as follows: 1 cycle at 95 °C for 2 min, 40 cycles of 95 °C for 15 s and 60 °C for 34 s, followed by a melting curve program (from 60 to 95 °C).

Twenty-three genes of interest were analyzed based on their key roles in placental functions: (1) genes encoding Arg transporters (solute carrier family 6 member 14 [*SLC6A14*], *SLC7A1*, *SLC7A6*, *SLC7A7*, *SLC7A9*); (2) genes encoding glucose transporters (*SLC2A1*, *SLC2A3*, *SLC2A4*); (3) genes encoding fatty acid transporters (*SLC27A1*, *SLC27A2*, *SLC27A3*); (4) genes associated with angiogenesis (vascular endothelial growth factor A [*VEGFA*], nitric oxide synthase 3 [*NOS3*], guanylate cyclase 1, soluble, beta 3 [*GUCY1B3*], hypoxia-inducible factor 1, alpha subunit [*HIF1A*]), and (5) genes involved in the mTOR pathway (serine/threonine-protein kinase 1 [*AKT1*], *mTOR*, ribosomal protein S6 kinase B1 [*RPS6KB1*], eukaryotic translation initiation factor 4B pseudogene 1 [*EIF4BP1*], *EIF4EBP2*, eukaryotic translation elongation factor 1 alpha 1 [*EEF1A1*], E74-like factor 2 [*ELF2*], insulin receptor substrate 1 [*IRS1*]). In addition, two references genes (glyceraldehyde-3-phosphate dehydrogenase [*GAPDH*] and beta-actin [*ACTB*]) were included based on recommendations from a previous study (Batistel et al., 2017). The primers targeting some of these genes were adapted from previous studies, whereas others were designed using the National Center for Biotechnology Information Nucleotide database (http://www.ncbi.nlm.nih.gov/nuccore/), with *Bos taurus* as the species (Appendix Table 1). The specificity of the primers was validated by primer-BLAST (https://www.ncbi.nlm.nih.gov/tools/primer-blast/). The relative changes at the mRNA level for each individual gene were analyzed using the 2^−ΔΔCT^ method, where ΔCT = CT _target mRNA_ – CT _housekeeping mRNA_, and CT is cycle threshold.

### 2.4 Metabolomics analysis

Fifty microliters of plasma sample were transferred into a 2-mL tube, and 200 μL pre-chilled extraction mixture (methanol) and 5 μL internal standard (L-2-chlorophenylalanine, 1 mg/mL stock) were added. The mixture was then vortexed for 30 s. The samples were then ultrasonicated for 10 min in ice water and centrifuged at 4 °C for 15 min at 16,065 × *g*. To prepare the quality control (QC) sample, 20 μL of each sample was collected and pooled together. After evaporation in a vacuum concentrator, 30 μL of methoxyamination hydrochloride (20 mg/mL in pyridine) was added. The mixture was then incubated at 80 °C for 30 min and then derivatized by 40 μL of bis(trimethylsilyl)trifluoroacetamide (BSTFA) regent (1% trimethylsilyl chloride, vol/vol) at 70 °C for 1.5 h. After gradually cooling the samples to room temperature, 5 μL of fatty acid methyl esters (FAME) (in chloroform) was added to the QC sample. All samples were then analyzed by a GC coupled with a time-of-flight mass spectrometer (GC-TOF-MS).

For the metabolomics, raw data processing, including peak extraction, baseline adjustment, deconvolution, alignment and integration (Kind et al., 2009), were performed with Chroma TOF (V 4.3x, LECO) software, and the LECO-Fiehn Rtx5 database was used for metabolite identification by matching the mass spectrum and retention index. Finally, the peaks detected in fewer than half the QC samples or RSD＞30% in the QC samples were removed (Dunn et al., 2011).

### 2.5 Statistical analysis

The pattern recognition multivariate analyses, including principal component analysis (PCA) and orthogonal partial least squares discriminant analysis (OPLS-DA), were performed with SIMCA 14.1 software package (V14.1, Sartorius Stedim Data Analytics AB, Umea, Sweden) with log transformation and the unit variance scaling conversion mode. The significantly different metabolites (SDM) were defined based on the variable importance for the projection (VIP) > 1.0 and *P* value < 0.05 (Sun et al., 2015). The SDM were further identified and validated by searching the online Kyoto Encyclopedia of Genes and Genomes (KEGG). Bovine Metabolome Database (BMDB). Metaboanalyst 3.0 (http://www.metaboanalyst.ca/) was employed to identify relevant pathways. The *B. taurus* (cow) pathway library was applied in this procedure.

The BW, blood parameters, AA composition and mRNA abundance of the placentome were analyzed using SAS software (version 9.0) according to following MIXED model procedure:

$$Y_{ij}=\mu +b_{i}+M_{j}+e_{ij}\text{,}$$

where *Y*_*ij*_ = dependent variable, *μ* = over-all mean, *b*_*i*_ = fixed effect of block (BW, blood parameters and AA composition: *i* = 1 to 15; mRNA abundance: *i* = 1 to 6), *M*_*j*_ = fixed effect of treatment (*j* = CON vs. NCG), *e*_*ij*_ = residual error. Significance was declared at *P* ≤ 0.05, and 0.05 < *P* ≤ 0.10 was considered as a trend.

## 3 Results

### 3.1 Birth weight, plasma parameters and AA composition of newborn calves

**Fig. 1:**
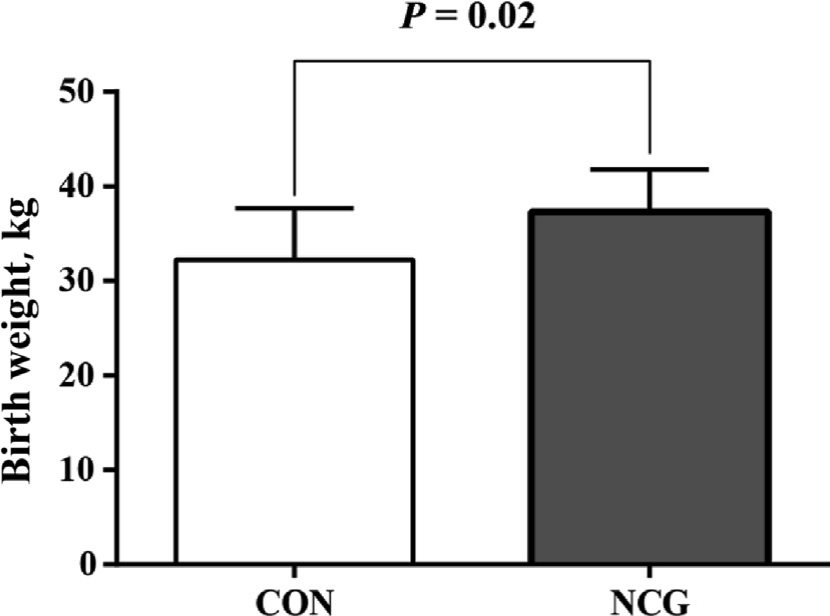
Birth weight of newborn calves from dairy cows supplemented with 0 (CON) or 20 g/d of *N*-carbamoylglutamate (NCG) during late gestation. Error bars indicate the SEM.

The gestation length of experimental dairy cows was 274.6 d (SD = 2.80) and 279.8 d (SD = 6.49) in the CON and NCG groups, respectively; but the difference was not statistically significant between the two groups (*P* = 0.22). Birth weight of calves in the NCG group was higher than that in the CON group (37.3 ± 1.28 kg vs. 32.3 ± 1.37 kg; *P* = 0.02, mean ± SEM, Fig. 1). The plasma concentration of urine nitrogen in calves tended to be lower (*P* = 0.10, Table 1) and plasma concentration of Arg was higher (*P* = 0.05, Table 2) in the NCG group than in the CON group. No differences were observed in the other tested plasma parameters between the two groups (Table 1, Table 2).

**Table 1:** Blood parameters of newborn calves from dairy cows supplemented without (CON) or with *N*-carbamoylglutamate (NCG) at 20 g/d during late gestation.

Item	CON	NCG	SEM	*P*-value
Glucose, mmol/L	3.31	2.67	0.40	0.29
Total protein, g/L	45.9	45.3	1.27	0.77
BUN, mmol/L	3.22	2.85	0.19	0.10
NEFA, μmol/L	650	399	190	0.39
BHB, μmol/L	37.4	38.4	3.89	0.87
Cholesterol, mmol/L	0.61	0.65	0.06	0.68
Triglyceride, mmol/L	0.16	0.10	0.03	0.26
Total bilirubin, μmol/L	6.00	8.05	2.50	0.58
Creatinine, μmol/L	203	220	27.4	0.68
Albumin (A), g/L	23.6	23.8	0.42	0.65
Globulin (G), g/L	22.3	21.6	0.97	0.64
A-to-G ratio	1.02	1.12	0.05	0.20

BUN = blood urea nitrogen; NEFA = non-esterified fatty acid; BHB = β-hydroxybutyrate.

**Table 2:** Plasma amino acid (AA) composition of newborn calves from dairy cows supplemented without (CON) or with *N*-carbamoylglutamate (NCG) at 20 g/d during late gestation (μmol/L).

Item	CON	NCG	SEM	*P*-value
Essential AA				
Arg	62.9	77.6	5.04	0.05
Thr	533	511	43.6	0.73
Val	172	200	27.4	0.50
Met	18.9	29.6	6.88	0.30
Ile	39.7	56.4	13.2	0.40
Leu	65.9	79.8	17.7	0.60
Phe	160	179	8.84	0.17
Lys	50.4	82.1	23.7	0.35
His	100	118	13.4	0.40
Sub-total	1,204	1,259	79.3	0.64
Non-essential AA				
Ala	523	737	175	0.42
Cys	12.6	17.6	3.27	0.32
Try	28.6	26.9	3.54	0.74
Glu	122	159	19.8	0.23
Gly	732	745	41.6	0.83
Asp	42.5	47.6	4.16	0.42
Ser	118	127	10.4	0.49
Pro	157	275	38.6	0.44
Sub-total	1,736	2,135	215	0.44
Total AA	2,940	3,404	283	0.47

### 3.2 Gene expression of the placentome

**Fig. 2:**
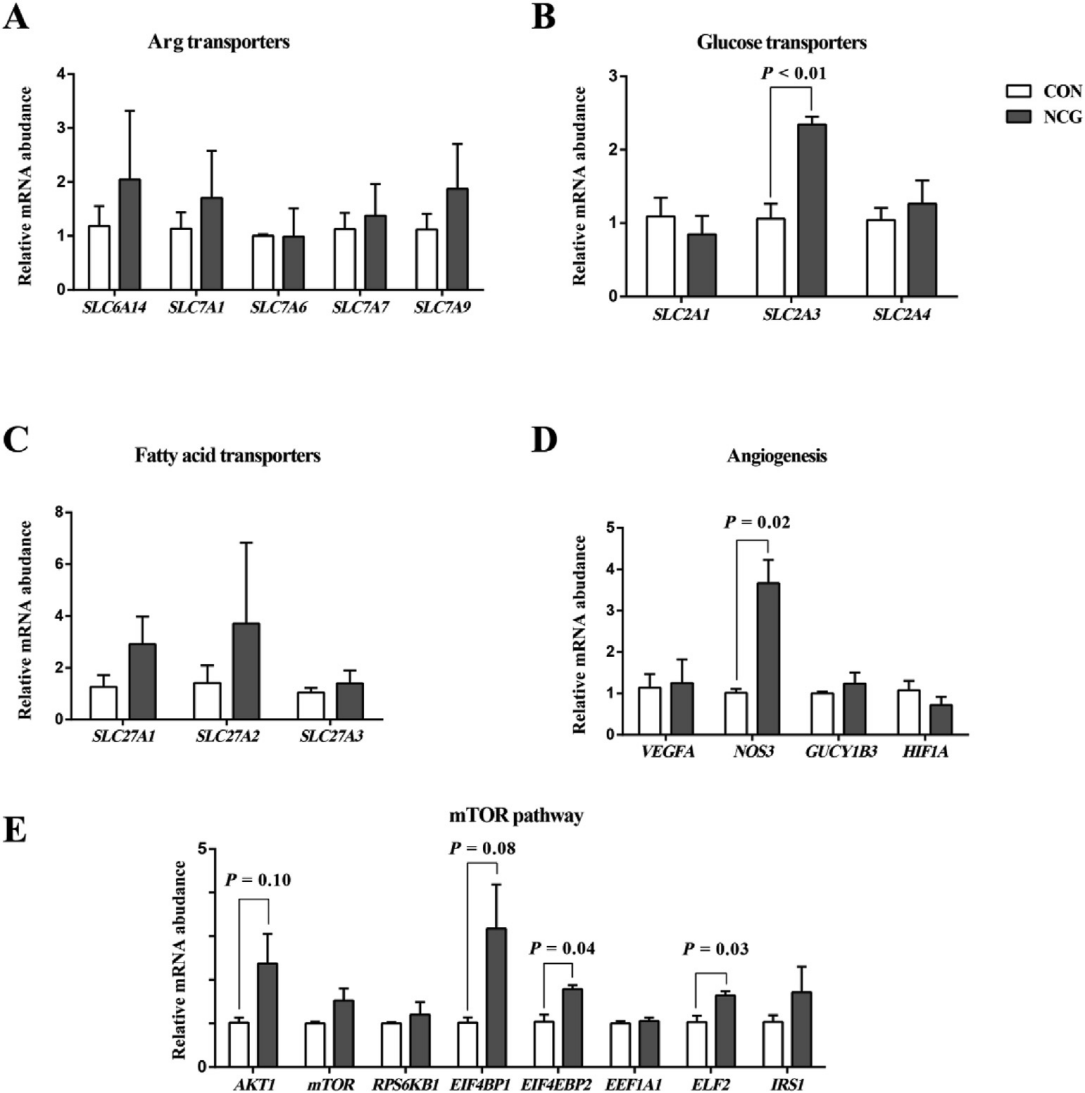
The mRNA abundance of Arg transporters (A), glucose transporters (B), fatty acid transporters (C), angiogenesis factors (D) and mTOR signaling molecules (E) in placentome of dairy cows supplemented without (CON) or with *N*-carbamoylglutamate (NCG) at 20 g/d during late gestation. Error bars indicate the SEM. The relative mRNA abundance for each individual gene was analyzed using the 2^−ΔΔCT^ method, in which ΔCT = CT _target mRNA_ – CT _housekeeping mRNA_, and CT is cycle threshold. *SLC6A14* = solute carrier family 6 member 14; *VEGFA* = vascular endothelial growth factor A; *NOS3* = nitric oxide synthase 3; *GUCY1B3* = guanylate cyclase 1, soluble, beta 3; *HIF1A* = hypoxia-inducible factor 1, alpha subunit; *AKT1* = serine/threonine-protein kinase 1; *mTOR* = mammalian target of rapamycin; *RPS6KB1* = ribosomal protein S6 kinase B1; *EIF4BP1* = eukaryotic translation initiation factor 4B pseudogene 1; *EEF1A1* = eukaryotic translation elongation factor 1 alpha 1; *ELF2* = E74-like factor 2; *IRS1* = insulin receptor substrate 1.

The mRNA expression of genes associated with Arg, glucose and fatty acid transporters, angiogenesis factors, and the mTOR signal pathway in the placentome are presented in Fig. 2. Supplementation of NCG during late gestation resulted in increased mRNA abundance of *SLC2A3* (*P* < 0.01) and *NOS3* (*P* = 0.02). The mRNA abundance of *AKT1* (*P* = 0.10) and *EIF4BP1* (*P* = 0.08) tended to be higher; and the mRNA abundance of *EIF4EBP2* (*P* = 0.04) and *ELF2* (*P* = 0.03) were higher in NCG group than in the CON group.

### 3.3 Calf plasma metabolite profiling using GC-TOF-MS

**Fig. 3:**
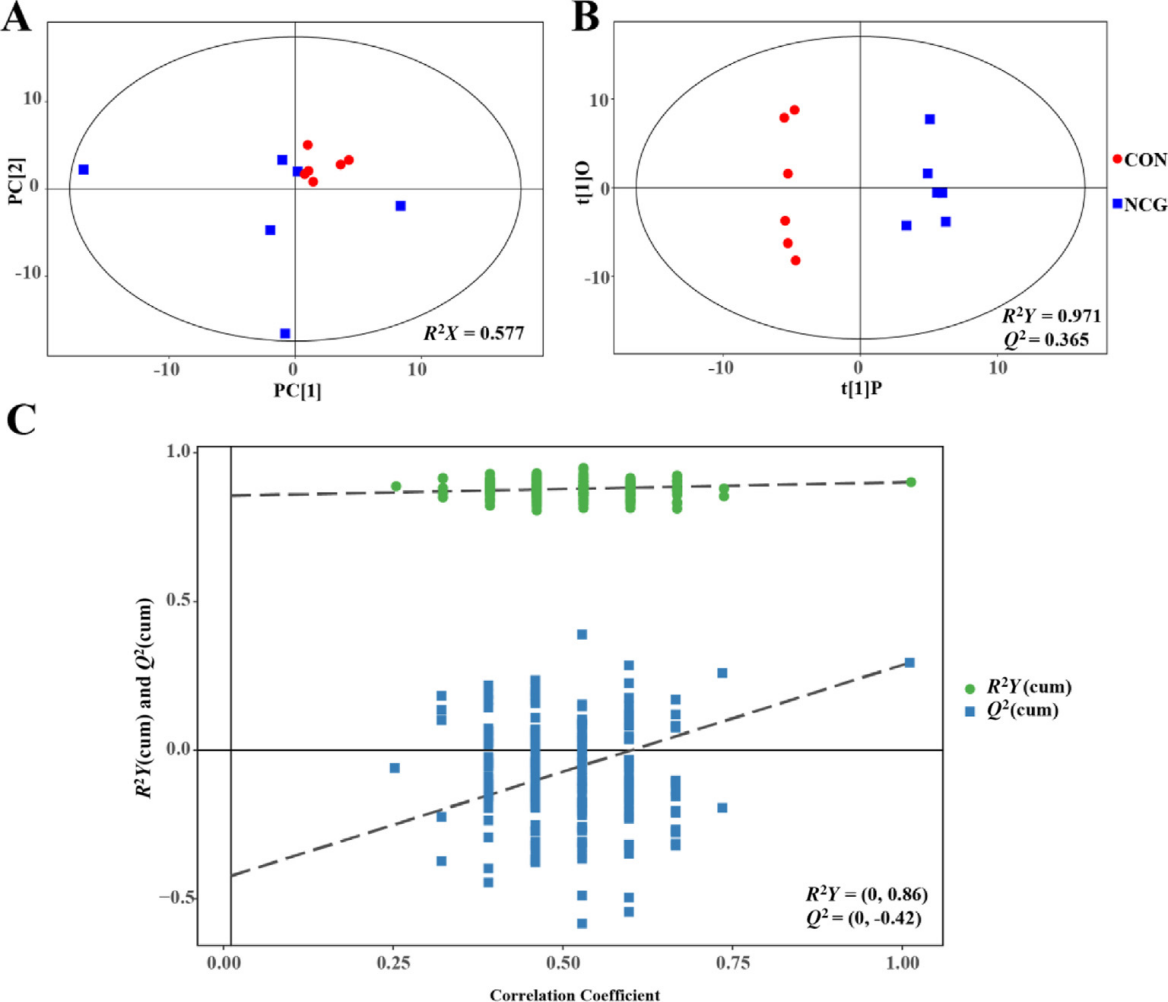
The PCA score map (A), OPLS-DA score plot (B), and corresponding validation plot of OPLS-DA (C) derived from the plasma metabolite profiles of calves of dairy cows supplemented without (CON, red circle) or with 20 g/d *N*-carbamoylglutamate (NCG, blue box) during late gestation. PCA = principal component analysis. OPLS-DA = orthogonal partial least squares discriminant analysis; PC = principal component.

Mass spectrometry showed a total of 303 effective peaks; 145 of them were identified and quantified, and 158 were labeled “analyte” or “unknown” based on analysis via the LECO-Fiehn Rtx5 database. The results of the multivariate analysis of metabolic profile differences between calves in the CON and NCG calves are shown in Fig. 3. No clear distinction was found between the two groups of calves according to the PCA score plot (Fig. 3A). In the PCA model, the R2X value was 0.577. The OPLS-DA score plot showed separated clusters between the two groups (Fig. 3B). Two evaluation parameters for the classification from the OPLS-DA model were calculated: *R*^2^*Y* = 0.971 and *Q*^2^ = 0.365. The results of permutations are presented in Fig. 3C, with intercept *R*^2^*Y* = (0, 0.86) and *Q*_2_ = (0, −0.42), indicating that the OPLS-DA model was sufficiently robust for downstream analysis.

### 3.4 Identification of SDM and pathways

**Fig. 4:**
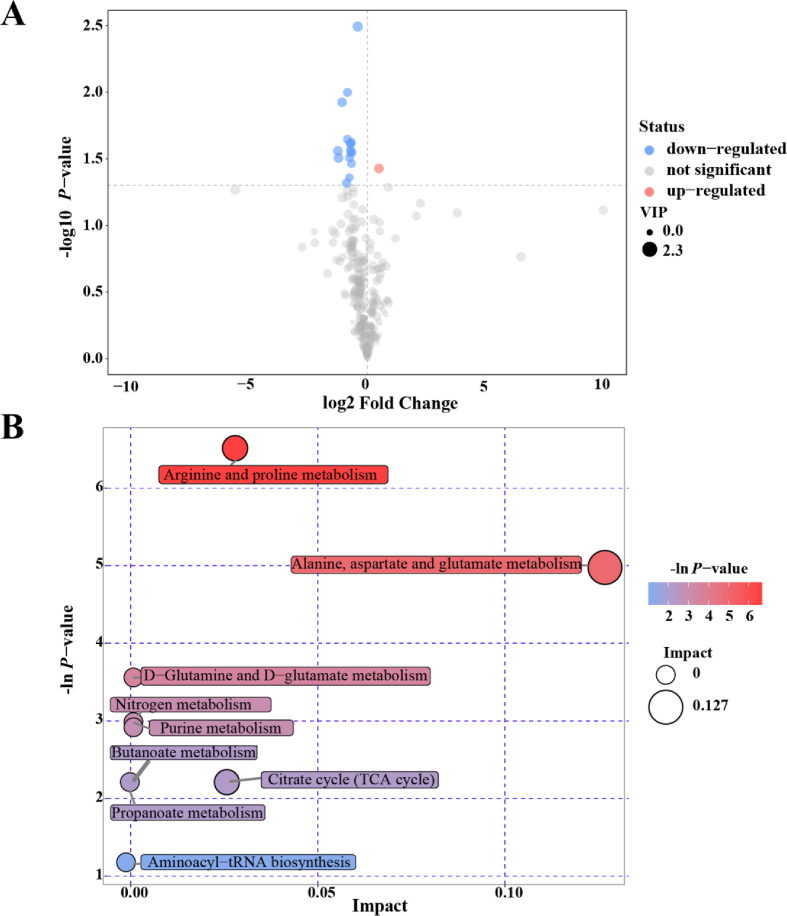
Volcano plot of serum metabolites (A) and metabolome view map of the significantly different metabolites (B) identified in calves from the dairy cows supplemented with 0 (CON) or 20 g/d of *N*-carbamoylglutamate (NCG) during late gestation. In the A, the x-axis represents log2 (FC) value, and the y-axis means - log10 (*P*-value). The red dot indicates the significantly different metabolite (SDM) that are more abundant in the NCG group, the blue dot represents the SDM with higher concentration in the CON cows. The dot size represents the variable importance in the projection (VIP) value. FC = fold change, mean value of peak area obtained from the NCG group/mean value of peak area obtained from the CON. In the B, the x-axis represents the pathway impacted, and the y-axis represents the pathway enrichment. Larger sizes and darker colors represent higher pathway impact values and higher pathway enrichment, respectively.

Seventeen SDM and 7 significantly different unidentified peaks were found between calves of the CON and NCG groups (Table 3; Fig. 4A). Among these SDM, citrulline [Log2 (FC) = 0.49, where FC is fold change] was more abundant in the NCG group, whereas 9 SDM were enriched in the CON group, including three metabolites in AA metabolism, i.e., urea [Log2 (FC) = −0.76], Gln [Log2 (FC) = −0.63] and *N*-acetyltryptophan [Log2 (FC) = −0.71]; two organic acids, i.e., succinic acid [Log2 (FC) = −0.87] and cyclohexyl sulfamic acid [Log2 (FC) = −0.70]; and four alcohols, i.e., cholestane-3,5,6-triol [Log2 (FC) = −1.06], phytol [Log2 (FC) = −0.66], 1-hexadecanol [Log2 (FC) = −0.71] and citraconic acid [Log2 (FC) = −1.24].

**Table 3:** Significantly different metabolites in plasma of newborn calves from cows supplemented with or without 20 g/d of *N*-carbamoylglutamate during late gestation.

Peak	Similarity	VIP	*P*-value	Fold change^1^
Succinic acid	898	1.57	0.048	0.546
Citrulline	714	1.91	0.037	1.407
Urea	529	1.14	0.044	0.592
Cholestane-3,5,6-triol (3β, 5α, 6β)	394	1.79	0.012	0.478
Unknown	388	1.30	0.028	0.641
Glutamine	309	1.31	0.024	0.630
Analyte	293	1.43	0.028	0.616
Unknown	285	2.34	0.003	0.757
1-Hexadecanol	285	1.34	0.027	0.612
Phytol	280	1.33	0.034	0.630
Citraconic acid	246	1.93	0.028	0.422
Analyte	236	1.46	0.010	0.560
Analyte	230	1.42	0.031	0.596
Cyclohexylsulfamic acid	227	1.38	0.024	0.614
Analyte	226	1.92	0.031	0.430
*N*-Acetyltryptophan	209	1.51	0.023	0.561
Unknown	197	1.40	0.024	0.608

VIP = variable importance for the projection.

1 The ratio of the concentration of metabolites in plasma of newborn calves from cows supplemented with to without 20 g/d of *N*-carbamoylglutamate.

Nine SDM-enriched metabolic pathways were identified and are illustrated in a metabolome view map (Table 4; Fig. 4B). These SDM were mainly involved in the following pathways: Arg and Pro metabolism (*P* = 0.001, impact value = 0.027); Ala, Asp and Glu metabolism (*P* = 0.007, impact value = 0.127); and TCA cycle (*P* = 0.109, impact value = 0.01).

**Table 4:** Significantly different pathway in plasma of new calves from cows supplemented with or without 20 g/d of *N*-carbamoylglutamate during late gestation.

Pathway	Total	Hits	Raw *P*-value	FDR	Impact
Arginine and proline metabolism	44	3	0.001482	0.12002	0.02785
Alanine, aspartate and glutamate metabolism	23	2	0.006896	0.2793	0.12667
D-Glutamine and D-glutamate metabolism	5	1	0.028468	0.76863	0
Nitrogen metabolism	9	1	0.050729	0.88268	0
Purine metabolism	68	2	0.054486	0.88268	0
Propanoate metabolism	20	1	0.10966	1	0
Butanoate metabolism	20	1	0.10966	1	0
Citrate cycle (TCA cycle)	20	1	0.10966	1	0.02566
Aminoacyl-tRNA biosynthesis	64	1	0.31463	1	0

FDR = false discovery rate.

## 4 Discussion

Our study found that the birthweight in the NCG group was 5.0 kg higher than in the control. Gestation length is one of the influencing factors of birthweight in dairy cows (Holland and Odde, 1992). However, no significant difference of gestation was found between the two groups in the current study. It is reported that fetal growth rate at the time of approaching parturition varied from 100 to 250 g/d (Eley et al., 1978; Prior and Laster, 1979; Holland et al., 1990), thus 5-d longer gestation would not cause such a big difference of birthweight (32.3 vs. 37.3 kg) observed in the current study.

In our previous companion study, dietary NCG supplementation increased the AA supply and improved the health status of transition dairy cows (Gu et al., 2021). The nutrient supply and maternal health are closely related to fetal growth and development (Tao et al., 2012). Arg is one of the most versatile AA and plays multiple physiological functions in animals (Morris, 2009; Tan et al., 2012). The placenta is the channel through which the fetus obtains nutrients and oxygen from the mother and plays important roles in the growth and development of the fetus. Maternal nutrient availability and placental transport efficiency are major intrauterine factors influencing fetal development (Brett et al., 2014). Adequate angiogenesis of the placenta is also important for providing adequate maternal nutrients and blood flow to the fetus. As a precursor of nitric oxide (NO), Arg could enhance placental angiogenesis (Cai et al., 2018; Raghavan and Dikshit, 2004). In the current study, *NOS3*, an important angiogenesis factor (Wu et al., 2012), was expressed higher in the placenta of dairy cows receiving NCG than in the CON cows, indicating that placental vasculogenesis and angiogenesis may be enhanced by NCG supplementation. This effect of NCG is consistent with the increase in NO concentration (Gu et al., 2021), and contributes to enhancing the delivery of nutrients and oxygen to the fetus, promoting fetal growth and development.

Fetal access to maternal nutrients requires the involvement of nutrient transporters in the placenta. In the current study, the plasma Arg concentration was higher in the newborn calves from cows receiving NCG than cows without NCG supplementation. No change was found in the Arg transporter of placenta, thus the higher Arg content in NCG newborn calves may be attributable to higher plasma Arg contents in their mothers (70.2 vs. 54.2 μmol/L; Gu et al., 2021). Interestingly, the expression of glucose transporter (*SLC2A3*) was enhanced in the placentome of the NCG group relative to the CON group. It has been reported that NO stimulates expression of glucose transporter in human placenta (Acevedo et al., 2005). In addition, maternal supplementation of Pro may increase the abundance of glucose transporter (*SLC2A3*) mRNA in placenta of mice (Liu et al., 2019). Both NO and Pro are important metabolites of Arg metabolism and were higher in the NCG supplemented dairy cows in the previous companion study (Gu et al., 2021), which may be attributable to the increased glucose transporter expression. Therefore, the higher expression of *SLC2A3* of placenta may be due to the increased Arg concentration with NCG supplemented cows. As the main source of energy, glucose is required for fetal growth and metabolism. Bell (1995) and Gao et al. (2009) reported that fetal gluconeogenesis is marginal in cattle, and the fetus is mainly dependent on glucose from maternal circulation. The transport of glucose and other polyols, such as fructose, galactose, mannose, and maltose, in the placenta is mediated by facilitated diffusion down a concentration gradient through the members of the glucose transporters (GLUT) family, such as GLUT1 (*SLC2A1*) and GLUT3 (*SLC2A3*) (Baumann et al., 2002). These transporters have a heterogeneous distribution across the placental membrane (Baumann et al., 2002). For example, GLUT1 is mainly located in the basal membrane, whereas GLUT3 is predominant in the microvillus membrane; the sequential action of both transporters is involved in glucose transport to the fetal circulation (Wooding et al., 2005). Therefore, the increased expression of *SLC2A3* observed in NCG cows indicates that the change in glucose transport mainly occurred in the microvillus membrane rich in capillaries, and this increase may be associated with the increased placental angiogenesis.

In addition to nutritional transport activity to affect the acquisition of maternal nutrients by the fetus, the placenta tissue can sense nutrient levels through nutrient-sensing signaling pathways, such as the mTOR complex (Jansson et al., 2013). This complex is a Ser/The protein kinase that may regulate cell growth and proliferation via activation of the ribosomal protein S6 kinase to phosphorylate ribosomal protein S6 and consequently regulate protein synthesis and other processes (Hay and Sonenberg, 2004). Nutrient levels, especially those of AA, represent major upstream regulators of mTOR. The activation of mTOR in the placenta determines fetal growth, and placental mTOR constitutes a mechanistic link between maternal nutrient availability and fetal growth (Roos et al., 2009). Batistel et al. (2017) reported that supplemental Met during late gestation increases feed intake and BW in newborn calves, possibly via the involvement of mTOR signaling in the placenta. Zhang et al. (2019) reported that Arg can help mitigate the negative effect of intrauterine growth restriction on nutrient absorption in neonatal lambs by regulating the mTOR signaling pathway. Glucose and individual AA, such as Leu and Arg, can independently regulate mTOR signaling (González et al., 2012). Therefore, the enhanced mRNA abundance of genes associated with mTOR signaling observed in the NCG group may be attributed to the increased Arg concentration. This phenomenon may contribute to the delivery of maternal nutrients to the fetus, leading to improved fetal growth.

Metabolomic analysis of the plasma of calves indicated that maternal NCG supplementation increased fetal plasma citrulline levels and improved AA metabolism and the citrate cycle. These findings are consistent with the higher Arg concentration in newborn calves from NCG supplemented cows and our previous finding in transition dairy cows that Arg levels increased under NCG supplementation (Gu et al., 2021). Arg plays a key role in ureagenesis as a precursor of citrulline during the urea cycle (Meijer et al., 1985). Therefore, these results indicate that the fetuses of NCG-supplemented cows may have obtained more maternal Arg than the fetuses of CON cows, and thus experienced the increased citrulline synthesis and urea cycling, as reflected in their lower levels of blood urea nitrogen.

## 5 Conclusions

Supplementation of NCG in dairy cows during late gestation increases the birth weight of the newborn calves. This effect of NCG supplementation may be attributed to the increased supply of nutrients to the fetus from the mother, mediated by increased uteroplacental angiogenesis, enhanced glucose transportation, and activated mTOR signaling in the placenta, as well as the increased blood concentration of AA (Arg and citrulline) in calves.

## Author contributions

**Fengfei Gu:** Methodology, investigation, data curation, writing - original draft preparation; **Luyi Jiang:** Investigation, data curation; **Linyu Xie:** data curation, investigation; **Diming Wang:** Project administration, writing - reviewing and editing; **Jianxin Liu:** Funding acquisition, supervision, visualization, project administration, writing - reviewing and editing.

## Conflict of interest

We declare that we have no financial and personal relationships with other people or organizations that can inappropriately influence our work, and there is no professional or other personal interest of any nature or kind in any product, service and/or company that could be construed as influencing the content of this paper.
