# Supplementary material for: Supplementing N-carbamoylglutamate in late gestation increases newborn calf weight by enhanced placental expression of mTOR and angiogenesis factor genes in dairy cows
Source: Anim Nutr. 2021 Sep 28;7(4):981–8. doi: 10.1016/j.aninu.2021.05.007 (PMC8551415; doi:10.1016/j.aninu.2021.05.007)
Supplement: Multimedia component 1 [file mmc1.doc]

**Appendix Table 1**

Primers used in quantitative PCR analysis

| Gene | NCBI accession no. | Sequence^1^ | Amplicon length (bp) | References |
| --- | --- | --- | --- | --- |
| Target genes | | | | |
| *SLC6A14* | NM_001098461.1 | F: GACGCCAACAATCTTACC | 182 | This study |
|  |  | R: AATGCTGCTCCGACTATG |  |  |
| *SLC7A1* | NM_001135792.1 | F: TCTGCTACGGCGAGTTTG | 483 | This study |
|  |  | R: CGGGTTTCCCTTCCTTTGT |  |  |
| *SLC7A6* | XM_024979000.1 | F: TTTGCCAACTACATCATCC | 271 | This study |
|  |  | R:AGAGGTCTCCCACATCCC |  |  |
| *SLC7A9* | XM_024978048.1 | R: ATACGCCCAGAGTCCATT | 345 | This study |
|  |  | R: GCCCAGGATGTGTAGCCTTT |  |  |
| *SLC7A7* | NM_001075151.1 | F: TCGGGCATCTTTGTCTCC | 365 | This study |
|  |  | R: ATGGGCTGCACCAGGTAG |  |  |
| *SLC2A1* | M60448 | F: CCCCCAGAAGGTGATTGAAG | 135 | Moyes et al., 2014 |
|  |  | R: GAACCAATCATGCCTCCCAC |  |  |
| *SLC2A3* | NM_174603.3 | F:CTCGGCCGCGTTCTACTTA | 117 | Batistel et al., 2017 |
|  |  | R:TCCTCAAAAGTCCTGCCACG |  |  |
| *SLC2A4* | XM_024977054.1 | F: CAGGTGCTAGGTTTGGAGTC | 159 | This study |
|  |  | R: GTGGCATAGGCTGGTTTCT |  |  |
| *SLC27A1* | NM_001033625.2 | F: GAGGGCTTCAGGGTCTCTAGGAT | 199 | Batistel et al., 2017 |
|  |  | R: CGGAAAGGCCGAAGAGGTCC |  |  |
| *SLC27A2* | NM_001192863.1 | F: CGGAGGAATGAGTCAGACGG | 151 | Batistel et al., 2017 |
|  |  | R: CCCTTTCCACCGGAATGTGT |  |  |
| *SLC27A3* | XM_015462580.1 | F: TCCAGCCTGGGGATGTTTTC | 116 | Batistel et al., 2017 |
|  |  | R: CACATTCTCCCCCTTCCACC |  |  |
| *VEGFA* | NM_174216.2 | F: GCAAGGCAAGAAAATCCCTGTG | 164 | This study |
|  |  | R: CGCCTCGGCTTGTCACATCT |  |  |
| *NOS3* | NM_181037.3 | F: GCCAAGCGAGTGAAAGCAA | 194 | This study |
|  |  | R: GGATCGCCATTCCCAAAG |  |  |
| *GUCY1B3* | NM_174641.1 | F: GTCTGTCTTCTCCCTGGTC | 112 | This study |
|  |  | R: CTCTACATCCAACAACCCT |  |  |
| *HIF1A* | NM_174339.3 | F: GGTATTATTCAGCACGAC | 351 | This study |
|  |  | R: CTTTGGAGTTTCAGAGGC |  |  |
| *AKT1* | NM_173986.2 | F: CTGCACAAGCGAGGTGAGTA | 134 | Batistel et al., 2017 |
|  |  | R: GAGAAGTTGTTGAGGGGCGA |  |  |
| *mTOR* | NM_001144096.3 | F: GGTTAACACCAAGCAGGTTCAT | 134 | Batistel et al., 2017 |
|  |  | R: GTATGTCGCACTGGACACCA |  |  |
| *RPS6KB1* | NM_205816.1 | F: ACAGCCTGCTTTTACTTGGC | 178 | Batistel et al., 2017 |
|  |  | R: AGGTGTGTGTGACTGTTCCG |  |  |
| *EIF4BP1* | NM_001077893.2 | F: GGAGTGTCGGAACTCACCTG | 162 | Batistel et al., 2017 |
|  |  | R: AACTGTGACTCTTCACCGCC |  |  |
| *EIF4EBP2* | NM_001191149.1 | F: AAGCATGCAGTTGGGGATGA | 158 | Batistel et al., 2017 |
|  |  | R: AACGGGGATTGCTGGTACTG |  |  |
| *EEF1A1* | NM_174535.2 | F: CCCTTCCTGGGGACAATGTG | 134 | Batistel et al., 2017 |
|  |  | R: AATAATCACCTGAGCTGTGAAGC |  |  |
| *ELF2* | NM_001191138.1 | F: GAGCTTCCCAGCAATGGAGT | 113 | Batistel et al., 2017 |
|  |  | R: GCTGCATAGCCCTGCTCTAA |  |  |
| *IRS1* | XM_003581871.4 | F: CTCAAGAGTGCCCACCTCAA | 187 | Batistel et al., 2017 |
|  |  | R: AGGTCTTCATTCTGCTGTGAT |  |  |
| Housekeeping genes | | | | |
| *GAPDH* | NM_001034034.2 | F: TTGTCTCCTGCGACTTCAACA | 103 | Garcia et al., 2016 |
|  |  | R: TCGTACCAGGAAATGAGCTTGAC |  |  |
| *ACTB* | NM_173979.3 | F: GCGGCATTCACGAAACTAC | 249 | Boulougouris et al.,2019 |
|  |  | R: TCTGCTGGAAGGTGGACAG |  |  |

^1^F=Forward, R=Reversed

**References**

Batistel F, Alharthi AS, Cardoso FC, Wang L, Parys C, Pan YX et al. Placentome nutrient transporters and mammalian target of rapamycin signaling proteins are altered by the methionine supply during late gestation in dairy cows and are associated with newborn birth weight. J Nutr 2017; 147: 1640 - 47.

Boulougouris X, Rogiers C, Van Poucke M, De Spiegeleer B, Peelma LJ, Duchateau L et al. Distinct neutrophil C5a receptor inflammatory events in cows initiated by chemoattractant C5a and lipopolysaccharide around parturition and in mid lactation. J Dairy Sci 2019; 102: 1457 - 72.

Garcia M, Elsasser TH, Juengst L, Qu Y, Bequette BJ, Moyes KM. Short communication: Amino acid supplementation and stage of lactation alter apparent utilization of nutrients by blood neutrophils from lactating dairy cows in vitro. J Dairy Sci 2016; 99: 3777 - 83.

Moyes, KM, Graugnard DE, Khan MJ, Mukesh M, Loor JJ. Postpartal immunometabolic gene network expression and function in blood neutrophils are altered in response to prepartal energy intake and postpartal intramammary inflammatory challenge. J Dairy Sci 2014*;* 97: 2165 - 77.
